# Advancements and challenges in immunocytokines: A new arsenal against cancer

**DOI:** 10.1016/j.apsb.2024.07.024

**Published:** 2024-08-02

**Authors:** Wenqiang Shi, Nan Liu, Huili Lu

**Affiliations:** a Shanghai Frontiers Science Center of Drug Target Identification and Delivery, National Key Laboratory of Innovative Immunotherapy, School of Pharmacy, Shanghai Jiao Tong University, Shanghai 200240, China; b Engineering Research Center of Cell & Therapeutic Antibody, Ministry of Education, School of Pharmacy, Shanghai Jiao Tong University, Shanghai 200240, China; c Shanghai Institute of Materia Medica, Chinese Academy of Sciences, Shanghai 201203, China

**Keywords:** Immunocytokines, Bifunctional fusion proteins, Combination therapy, Cytokine engineering, Tumor-conditional, Prodrug

## Abstract

Immunocytokines, employing targeted antibodies to concentrate cytokines at tumor sites, have shown potential advantages such as prolonged cytokine half-lives, mitigated adverse effects, and synergistic antitumor efficacy from both antibody and cytokine components. First, we present an in-depth analysis of the advancements of immunocytokines evaluated in preclinical and clinical applications. Notably, anti-PD-1-based immunocytokines can redirect cytokines to intratumoral CD8^+^ T cells and reinvigorate them to elicit robust antitumor immune responses. Then, we focus on their molecular structures and action mechanisms, striving to elucidate the correlations between diverse molecular structures and their antitumor efficacy. Moreover, our exploration extends to the realm of novel cytokines, including IL-10, IL-18, and IL-24, unraveling their potential in the construction of immunocytokines. However, safety concerns remain substantial barriers to immunocytokines' development. To address this challenge, we explore potential strategies, such as cytokine engineering and prodrug design, which can foster next-generation immunocytokines development. Overall, this review concentrates on the design of molecular structures in immunocytokines, underscoring the direction and focus of ongoing efforts to improve safety profiles while maximizing therapeutic efficacy.

## Graphical abstract

Immunocytokines delivering immunostimulatory payloads to tumor lesions can substantially broaden the therapeutic window of cytokine therapy. Engineering cytokine and prodrug strategies can address safety challenges of immunocytokines.

**Figure:**
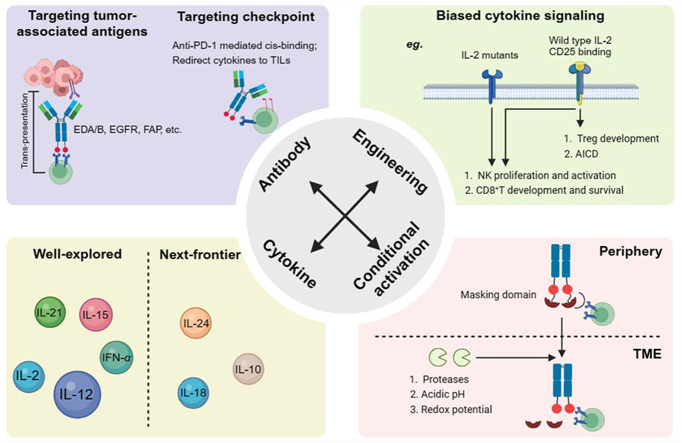

## 1 Introduction

**Figure 1:**
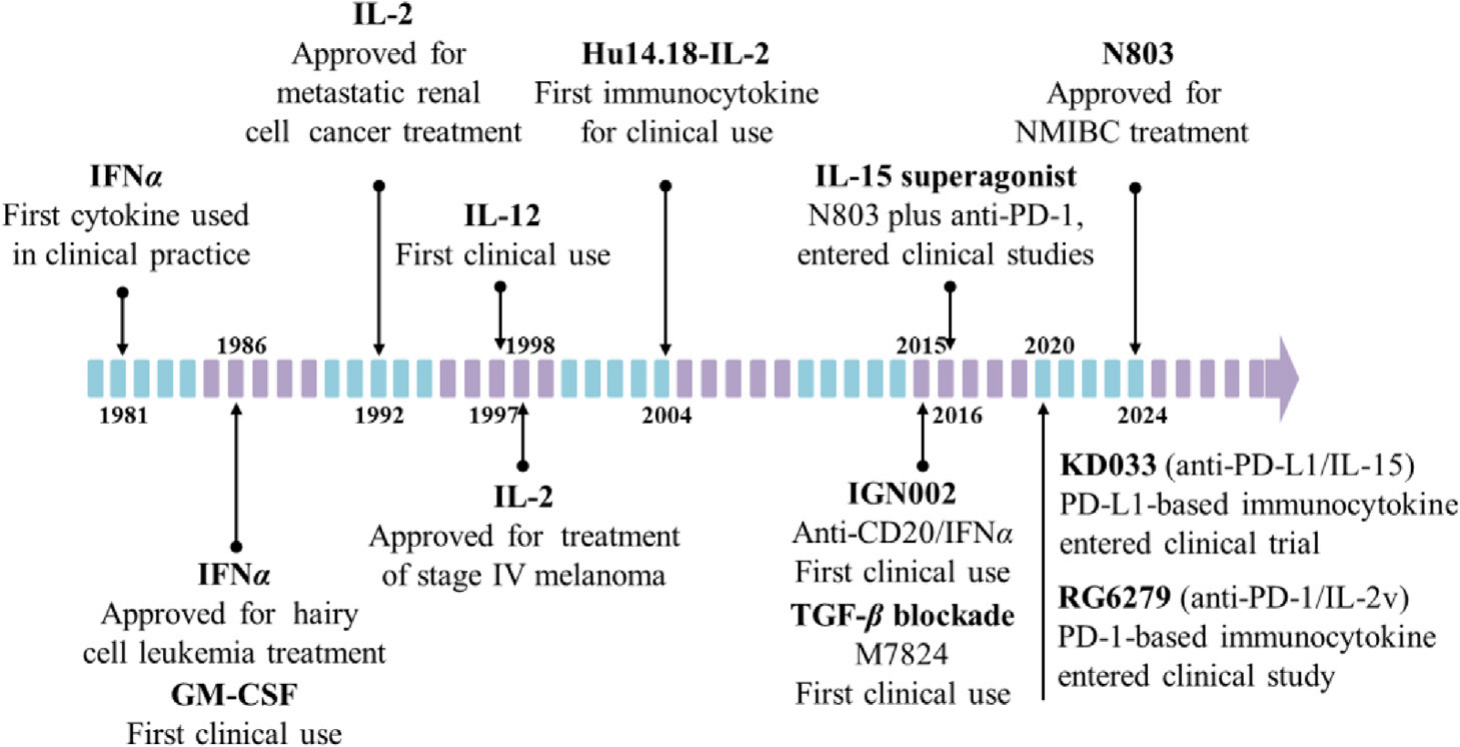
The history of cytokines and immunocytokines for cancer therapy^6^^,^^16^, ^17^, ^18^, ^19^, ^20^, ^21^, ^22^, ^23^.

Cytokines play a vital role in modulating immune responses and have the potential to regulate the tumor microenvironment (TME), thereby exhibiting potent antitumor effects^1^. Since the approval of interferon alpha (IFN-*α*) and interleukin-2 (IL-2) in 1986 and 1992 respectively, there has been a surge in research on other cytokines, such as IL-10, IL-12, IL-15, granulocyte-macrophage colony-stimulating factor (GM-CSF), transforming growth factor-*β* (TGF-*β*), etc.^2^, ^3^, ^4^, ^5^, ^6^, ^7^. However, due to concerns regarding efficacy and safety, they have yet to receive market approval. The clinical utilization of cytokines has long been limited by short half-life and off-target systemic toxicity, attributed to their high affinity for endogenous receptors which triggers functional signaling even at picomolar concentrations, leading to peripheral “cytokine sink” effects^8^^,^^9^. The breakthrough came in 2024 with the approval of N-803 (an IL-15 superagonist), marking only the third cytokine to be listed. But it is indicated for treating non-muscle invasive bladder cancer (NMIBC) combined with Bacillus Calmette-Guerin *via* intravesical instillation rather than systemic administration (NCT03022825) (Fig. 1).

A practical strategy is to employ antibodies to selectively deliver cytokines to tumor sites, thereby extending their half-life and reducing toxicity. These antibody-cytokine fusion proteins, known as immunocytokines, have shown great potential in research and development, with dozens of molecules advancing to clinical stages^2^. Analyzing Table 1 reveals a trend in clinical immunocytokine development. Earlier molecules in Phase II/III clinical trials predominantly target tumor-associated antigens^10^, ^11^, ^12^. However, recent developments in phase I clinical trials primarily target programmed death 1/programmed death-ligand 1 (PD-1/PD-L1), and the cytokine component diversifies, with a significant utilization of IL-15^13^^,^^14^. For instance, KD033, consisting of IL-15 fused to anti-PD-L1, demonstrated good tolerability in Phase I clinical trial^15^. This trend reflects the evolving landscape of immunocytokine research towards targeting specific immune checkpoints like PD-1/PD-L1.

**Table 1:** List of recent immunocytokines in clinical research.

Name	Target	Cytokine	Disease	Phase	Clinical trial
Hu14.18-IL-2	GD2	IL-2	Relapsed or refractory neuroblastoma Resectable recurrent stage III or stage IV melanoma	II	NCT00082758 NCT01334515 NCT00590824
FAP-IL2v	FAP	IL-2	Advanced and metastatic solid tumors	II	NCT03386721
F16-IL2	Tenascin-C	IL-2	Solid tumors	II	NCT02054884 NCT05468294
L19-IL-2	EDB	IL-2	Melanoma stage IIIB/C	III	NCT03567889
L19-TNF	EDB	TNF	Malignant melanoma	III	NCT02938299
NHS-IL-12	DNA/histones	IL-12	Metastatic solid tumors	I	NCT01417546
BC1-IL-12 (AS1409)	EDB	IL-12	Metastatic renal cell carcinoma Metastatic malignant melanoma	I	NCT00625768
RG6279	PD-1	IL-2	Advanced and metastatic solid tumors	I	NCT04303858
IBI363	PD-1	IL-2	Advanced solid malignancies or lymphomas	I	NCT05290597
KD033	PD-L1	IL-15	Metastatic or locally advanced solid tumors	I	NCT04242147
BJ-001	Integrin	IL-15	Locally advanced or metastatic solid tumors	I	NCT04294576
IAP0971	PD-1	IL-15	Advanced malignant tumors	I/IIa	NCT05396391
SIM0237	PD-L1	IL-15	Advanced solid tumors	I	NCT05781360
IGM-7354	PD-L1	IL-15	Relapsed and/or refractory tumors	I	NCT05702424
AMG256	PD-1	IL-21	Advanced solid tumors	I	NCT04362748

In this comprehensive review, we present an in-depth analysis of immunocytokines including their molecular structures, mechanisms of action, safety profiles, therapeutic effects, and other crucial characteristics. Our objective is to provide valuable insights into the potential applications and limitations of these fusion proteins, guiding the design and development of next-generation immunocytokines.

## 2 Prevalent cytokines for immunocytokines development

### 2.1 IL-2

IL-2 has been used to create various immunocytokines targeting fibronectin’s alternatively spliced extra domain A (EDA) or extra domain B (EDB), carcinoembryonic antigen (CEA), fibroblast activation protein (FAP), PD-1, and others^14^, as listed in Table 2. Notably, a combination therapy involving anti-EDB-IL-2 (L19-IL2) and anti-EDB-TNF (L19-TNF) has advanced to phase III trials to explore the efficacy of intratumoral treatment followed by surgery in clinical stage IIIB/C melanoma patients (NCT03567889 and NCT02938299). While wild type IL-2 was primarily used in earlier developed molecules like Hu14.18-IL2 and L19-IL2, newer molecules that employ mutated IL-2 with reduced affinity to IL-2R*α* or IL-2/15R*βγ* to enhance safety and efficacy are of increasing interest^2^^,^^24^^,^^25^.

**Table 2:** Representative IL-2-based immunocytokines.

Name	Structure	Target	Cytokine	Ref.
Anti-CEA-IL-2		CEA	IL-2	26
CBD-IL-2		Collagen	IL-2	29
PD-1-laIL-2		PD-1	• R38L, F42A • Reduced binding to IL-2R*α*/*β*	30
Erb-sumIL-2		EGFR	• F42A, L80F, R81D, L85V, I86V, I92F • Decreased binding to IL-2R*α* • Increased binding to IL-2R*β*	31
KY1043		PD-L1	• N-terminal truncation of IL-2 • Reduced binding to IL-2R*βγ*	32
CEA/FAP/PD1-IL2v		CEA/FAP/PD-1	• F42A, Y45A, L72G • IL-2R*βγ* binding • No binding to IL-2R*α*	33, 34, 35
IBI363		PD-1	• N88D • IL-2R*α* binding • Reduced binding to IL-2R*βγ*	24,36

#### 2.1.1 IL-2-based immunocytokines under preclinical research

Kujawski et al. developed a fully bioactive homodimeric immunocytokine, anti-CEA-IL-2, which comprises wild type IL-2 fused to the C-terminus of the clinically tested humanized anti-CEA antibody (hT84.66-M5A). In a mouse breast carcinoma model, combination therapy with anti-PD-1 antibody did not demonstrate improved tumor reduction compared to treatment with anti-CEA-IL-2 alone. However, it showed enhanced tumor inhibition and improved immunity against tumor rechallenge when combined with stereotactic tumor irradiation^26^.

Collagen is abundant presence in the tumor stroma. The hyperpermeability of tumor vasculature allows preferential interaction between circulating molecules and collagen within the tumor^27^. The A3 domain of von Willebrand factor (VWF) has been identified as a collagen-binding domain (CBD) with high affinity for collagen^28^. Hubbell team developed the CBD-fused IL-2 (CBD-IL-2) that exhibits tumor localization after intravenous administration. In contrast to unmodified IL-2, which can induce splenomegaly and pulmonary edema due to increased vascular permeability, CBD-IL-2 avoids these adverse effects. Moreover, CBD-IL-2 demonstrates superior efficacy in multiple cancer models compared to unmodified IL-2^29^.

Ren et al.^30^ engineered a low-affinity IL-2 with decreased binding to both IL-2R*α* and IL-2R*β* to fuse with anti-PD-1 (PD-1-laIL-2). Compared with PD-1-wtIL-2, PD-1-laIL-2 exhibits reduced affinity to peripheral regulatory T cells (Tregs), CD4^+^ T, and CD8^+^ T cells, allowing preferential targeting of intratumoral PD-1^+^CD8^+^ T cells. PD-1-laIL-2 displayed superior tumor inhibition and lower toxicity compared to single or combined treatments of anti-PD-1 and IL-2. Mechanistically, the antitumor efficacy of PD-1-laIL-2 relied on intratumoral CD8^+^ T cells, particularly PD-1^+^TIM3^+^ tumor-infiltrating lymphocytes (TILs)^30^.

Immunocytokines Erb-sumIL2 and Her2-sumIL2, featuring asymmetric structures, consist of a super mutant IL-2 (sumIL-2) with decreased IL-2R*α* binding and increased IL-2R*β* binding fused to epidermal growth factor receptor (EGFR) and human epidermal growth factor receptor 2 (Her2)-targeting antibodies, respectively^31^. In murine tumor models with high EGFR expression, Erb-sumIL2 significantly enhanced tumor control when compared with the non-EGFR targeting sumIL-2, while also inducing protective memory immunity. Mechanistically, the antitumor efficacy of Erb-sumIL2 relies on CD8^+^ T cells rather than natural killer (NK) cells. Besides, Her2-sumIL2 can synergize with targeted therapy to control cold tumors^31^.

KY1043 consists of a neutralizing anti-PD-L1 antibody fused to an IL-2 mutant featuring reduced affinity for IL-2R*βγ* through its light chains, thereby favoring binding to the high-affinity trimeric IL-2R*αβγ* receptor. It induced a potent and dose-dependent anti-tumor response and all mice survived when administered at a dosage of 10 mg/kg, with no significant weight loss observed. This unique design promotes proliferation of CD8^+^ T cells within the TME while also inducing a substantial expansion of Tregs in peripheral lymphoid tissue^32^. These results challenge the conventional belief that selective binding to the dimeric IL-2R*βγ* is essential for effective tumor control.

#### 2.1.2 IL-2-based immunocytokines under clinical research

Roche has developed an novel class of immunocytokines based on an engineered IL-2 variant (IL-2v) that retains affinity for IL-2R*βγ* while abolishing binding to IL-2R*α*. Examples include CEA-IL2v, FAP-IL2v, which exhibit enhanced tumor targeting abilities and improved pharmacokinetics compared to their wild-type counterparts^33^^,^^34^. Preclinical data indicates that both CEA-IL2v and FAP-IL2v may synergize with PD-L1 checkpoint blockade and antibody-dependent cellular cytotoxicity (ADCC) competent antibodies to yield potent antitumor effects. Moreover, in a phase I trial, CEA-IL-2v has been assessed in combination with atezolizumab for use against locally advanced and/or metastatic solid tumors (NCT02350673). A phase Ib/II study is underway to assess the effectiveness of combining FAP-IL-2v with other therapies for metastatic pancreatic ductal adenocarcinoma (NCT03193190). Recently, Roche has reported PD1-IL2v, which features cis binding to PD-1 and IL-2R*βγ* on the same cell, promoting the differentiation of stem-like CD8^+^ T cells into better effectors without requiring CD25 binding and providing superior efficacy^35^. It is currently in Phase I clinical trial (NCT04303858).

Research conducted by Innovent Biologics Inc. has shown that IL-2R*α*-biased agonists, compared to IL-2R*βγ*-biased agonists, significantly enhance tumor-specific CD8^+^ T cells (TSTs), demonstrating a superior antitumor efficacy. Additionally, IL-2R*α*-biased agonists exhibit a stronger stimulatory effect on peripheral Tregs, resulting in a better safety profile than their IL-2R*βγ-*biased counterparts. Moreover, PD-1^+^CD25^+^CD8^+^ TILs are enriched for TSTs^36^. Leveraging these insights, the Innovent developed an anti-PD-1/IL-2 fusion protein (IBI363), which could block PD-1 checkpoint and *cis*-activate the *α* subunit-biased IL-2 to revitalize exhausted TSTs. A phase I study demonstrated that IBI363 was well tolerated by all patients and showed encouraging efficacy in advanced non-small cell lung cancer (NSCLC) and melanoma (NCT05290597; NCT05460767). Notably, all squamous NSCLC patients at 3 mg/kg had partial response^24^.

### 2.2 IL-15

IL-15 plays a crucial role in the development, survival, and activation of NK cells and CD8^+^ T cells, and contributes to the homeostatic proliferation of memory CD8^+^ T cells, facilitating long-lasting immunity against tumors^37^. To date, IL-15 has been fused with various targeting antibodies or proteins, including TGF-*β* receptor ectodomain (TGF-*β*RII-ECD), anti-PD-1, and anti-PD-L1, as listed in Table 3.

**Table 3:** Representative IL-15-based immunocytokines.

Name	Structure	Target	Cytokine	Ref.
FIST15		TGF-*β*	Sushi-IL-15	38
N809		PD-L1	Sushi/IL-15 complex	39,40
LH01		PD-L1	Sushi-IL-15(N72D)	41
Anti-PD-1/IL15		PD-1	Sushi-IL-15	42
Anti-PD-1-IL15m		PD-1	• N1G, D30N, E46G, V49R, E64Q • Reduced affinity to IL-15R*βγ* • Elimination of IL-15R*α* binding	43
*α*PD-1-IL-15-R		PD-1	IL-15-Sushi	44
HCW9218		TGF-*β*	Sushi/IL-15 complex	45
KD033		PD-L1	Sushi-IL-15	46
SOT201		PD-1	• IL-15 mutein • Reduced affinity to IL-15R*βγ*	48
IAP0971		PD-1	Sushi/IL-15 complex	49
GTB-3550		CD16+CD33	IL-15 mutein (N72D)	52
Second-generation TriKEs		CD16+CD33/CLEC12A/Mesothelin	Wild type IL-15	54, 55, 56

#### 2.2.1 IL-15-based immunocytokines under preclinical research

FIST15 combines IL-15R*α*-sushi domain/IL-15 complex with two tandem TGF-*β* traps derived from portions of the TGF-*β*RII-ECD. In syngeneic mouse models, FIST15 demonstrated NK cell-dependent antitumor effects^38^.

N-809, a bifunctional fusion protein, comprises the IL-15/IL-15R*α* superagonist complex containing the Fc-domain of IgG1 fused to two single chain anti-PD-L1 domains. In murine breast and colon carcinoma models, N-809 treatments showed superior antitumor efficacy compared to non-targeting IL-15 plus anti-PD-L1. Mechanistically, the antitumor efficacy of N-809 is mediated by NK and CD8^+^ T cells, which are activated and function more effectively in the tumor-draining lymph node (dLN) and TME. N-809 also promotes the migration of NK cells and CD8^+^ T cells into the TME by regulating chemokine levels and chemokine receptor expression^39^^,^^40^.

Our laboratory has recently developed LH01, a novel immunocytokine consisting of anti-PD-L1 fused to IL-15R*α*-sushi domain/IL-15 complex. LH01 showed improved antitumor efficacy and safety *versus* the combination of anti-PD-L1 with non-targeting IL-15 superagonist and overcame resistance to anti-PD-L1 treatment. Mechanistically, LH01 activated both the innate and adaptive immune responses and induced an immunostimulatory TME^41^.

Kadmon developed four formats of novel immunocytokines containing IL-15 and anti-PD-1 antibodies, as shown in Table 3. Among these formats, the one with a single IL-15 fused to anti-PD-1 at the N-terminus demonstrated strong antitumor activity and effectively overcame resistance to PD-1 blockade^42^.

Pfizer reported an anti-PD1-IL15m immunocytokine, which consists of a single IL-15 mutein that lacks IL-15R*α* binding and has reduced affinity to IL-2R*βγ*, fused with a PD-1-specific antibody. Compared to the wild type anti-PD1-IL15, anti-PD1-IL15m demonstrated slower clearance in the serum due to decreased affinity to IL-2R*β*. Moreover, a single subcutaneous injection of anti-PD1-IL15m demonstrated robust dose-dependent efficacy, surpassing the combination of IL15 superagonist with anti-PD-1. However, treatment with at a 5 mg/kg dose of anti-PD1-IL15m led to body weight loss of tumor-bearing mice, attributed to upregulated peripheral NK cells. Mechanistically, anti-PD1-IL15m preferentially targeted CD8^+^ TILs and expanded an exhausted CD8^+^ TILs cluster with high proliferative capacity and effector-like signatures. Notably, the antitumor efficacy of anti-PD1-IL15m was found to rely on CD8^+^ T cells rather than NK cells^43^.

Shen et al.^44^ developed *α*PD-1-IL-15-R, a fusion protein consisting of anti-PD-1 and IL-15-IL-15R*α*, which showed remarkable antitumor efficacy without detectable toxicity across various kinds of tumor models. They used the Fc domain of *α*PD-1 antibody to shield IL-15 binding to IL-15R*β*, physically achieved by a delicate linker joining the Fc and IL-15. The extraordinary effectiveness of *α*PD-1-IL-15-R is attributed to the selective activation and expansion of PD-1^+^ tumor-specific CD8^+^ T cells *via cis*-delivery. Additionally, CD8^+^ T cells, but not NK or CD4^+^ T cells, played a crucial role in tumor control through IFN-*γ* secretion^44^.

#### 2.2.2 IL-15-based immunocytokines under clinical research

Building upon FIST15, HCW9218 was developed using a soluble tissue factor-based scaffold technology to combine TGF-*β*RII-ECD and IL-15R*α*-sushi domain/IL-15 complex, and displayed robust antitumor activity mediated by promoting the infiltration of NK cells and CD8^+^ T cells into tumors^45^. A phase I study indicated that HCW9218 can safely and robustly expands NK cells after a single dose and escalation continues to 0.5 mg/kg (NCT05322408).

Kadmon also reported KD033, a novel homodimeric immunocytokine composed of a fully human anti-PD-L1 linked to the IL-15R*α*-sushi domain/IL-15 complex. In various syngeneic murine tumor models, a single dose of KD033 demonstrated significant antitumor effects and good safety. KD033 activated both adaptive and innate immune cells within tumors, and the antitumor efficacy was largely dependent on CD8^+^ T cells rather than NK cells. Additionally, when combined with anti-PD-1, KD033 exerted synergistic antitumor activity^46^. In a phase I clinical trial, KD033 was well tolerated with expected and manageable adverse events^47^.

SOT201, a novel cis-acting immunocytokine, comprises a humanized, Fc-silenced anti-PD-1 antibody fused to a covalent sushi/IL-15 mutein complex with reduced IL-2R*βγ* affinity. *Via* cis presentation, SOT201 delivers attenuated sushi/IL-15 mutein to PD1^+^ TILs, stimulating *in vitro* exhausted T cells and expanding antigen-specific PD-1^+^CD8^+^ T cells *in vivo*. In PD-1 responsive and resistant tumor models, SOT201 demonstrated robust anti-tumor efficacy, outperforming a mouse PD-1-IL-2R*βγ* agonist. Studies in cynomolgus monkeys showed that decreased affinity of the novel IL-15 mutein in SOT201 for IL-2R*βγ* binding is well optimized to ensure favorable pharmacokinetic properties while potently stimulating PD-1^+^CD8^+^ T cells and NK cells^48^. SOT201 is currently in phase I clinical trial (NCT06163391).

IAP0971 is a heterodimeric fusion protein that binds specifically to human PD-1 and fuses the natural human cytokine IL-15/IL-15R*α* complex. It expanded CD8^+^ T cells and natural killer T cells (NKT) cells, and further activated NK cells to kill tumor cells validated by *in vitro* assays. In non-human primates, both single and repeated administrations of IAP0971 revealed a promising pharmacokinetic profile, along with a well-tolerated toxicity profile. The safety, tolerability, and preliminary efficacy of IAP0971 are currently being assessed in an ongoing phase I/IIa clinical trial (NCT05396391) for patients with advanced malignant tumors^49^.

#### 2.2.3 Tri-specific killer engagers (TriKEs)

NK-cell-based immunotherapies are quickly gaining traction due to the ability of NK cells to lyse tumors with minimal priming and their lack of MHC restriction^50^. Developing affordable NK-cell-based immunotherapeutic off-the-shelf products will significantly strengthen clinical practice. Compared to bispecific killer engager containing single chain variable fragments (scFv) against CD16 and tumor-associated antigen, TriKE further integrated with IL-15 exhibits a superior proliferation and activation of NK cells^51^.

Miller team incorporated a modified IL-15 cross-linker to construct a TriKE bearing scFv against CD16 on NK cells and CD33 on tumor cells (named GTB-3550), which can potently restore defective NK function and induce specific NK cell proliferation^52^. A phase I study demonstrates GTB-3550 TriKE safety, robust expansion of endogenous NK cells and a clinical signal of activity in acute myeloid leukemia (AML) and myelodysplastic syndromes patients (NCT03214666)^53^. Considering decreased IL-15 potency when sandwiched between the CD16 and CD33 scFvs, they further substituted the anti-CD16 scFv with a humanized single-domain antibody against CD16 and IL-15 mutant with wild type IL-15 to construct CD33-targeting second-generation TriKE^54^. It induced stronger and more specific NK-cell proliferation without T-cell stimulation and improved tumor control in preclinical mouse models. Additionally, they also developed a C-type lectin domain family 12 member A (CLEC12A)-targeting TriKE and mesothelin-targeting TriKE to treat AML and lung cancer, respectively^55^^,^^56^.

### 2.3 IL-12

IL-12 plays a crucial role in promoting the differentiation and proliferation of Th1 cells, reactivating and promoting the survival of CD4^+^ T cells, and enhancing the cytotoxicity of both CD8^+^ T cells and NK cells^57^. However, the clinical application of IL-12 faces dose-limiting toxicities, with a maximum tolerated dose of rhIL-12 reported as only 500 ng/kg in a phase I trial^2^^,^^58^. To address this challenge, various attempts, including the fusion of IL-12 with antibodies to create immunocytokines, have been explored (Table 4).

**Table 4:** Representative IL-12-based immunocytokines.

Name	Structure	Target	Cytokine	Ref.
IL12-F8-F8		EDA	IL-12	59
Split IL-12		EDA	p35(C92S) p40(C197S)	60
IL-12-MSA-Lumican		Collagen	IL-12	61
CBD-IL-12		Collagen	IL-12	62
*α*PD1-mIL12mut2		PD-1	p40(E81A; F82A)	63
NHS-IL12		DNA/DNA-histone complexes	IL-12	64
L19-IL12		EDB	IL-12	66
AS1409		EDB	IL-12	67

#### 2.3.1 IL-12-based immunocytokines under preclinical research

Pasche et al.^59^ developed IL-12-F8-F8, an immunocytokine based on the sequential fusion of IL-12 as a single polypeptide with two F8 antibodies in single-chain antibody fragment (ScFv) format, targeting the alternatively spliced EDA of fibronectin. It effectively inhibited tumor growth of F9 embryonal carcinoma, CT26 colon cancer, and A20 lymphomas. Notably, a synergistic antitumor effect combined with paclitaxel was observed only in the F9 embryonal carcinoma model. Further investigation revealed that NK cells primarily contributed to the antitumor efficacy mediated by IL12-F8-F8.

Considering that the intermolecular disulfide bond is not necessary to form active IL-12 heterodimers, Venetz et al. engineered p40 (C197S) and p35 (C92S) fused to irrelevant antibody and F8 antibody, respectively, allowing selective reassembly after binding to the target antigen *in vitro*^60^. The concept of stepwise antibody-based reassembly of split cytokines holds promise for the development of anticancer therapeutics with improved safety and tolerability.

Wittrup team has pioneered a method leveraging the abundance of collagen in tumors to enhance the retention of injected cytokine fusion proteins. This involves fusing cytokines with lumican, a collagen-binding protein, thereby ensuring their confinement upon intratumoral injection. Intratumorally administered collagen-anchoring IL-12 demonstrated prolonged retention and effectively eliminated systemic exposure toxicity compared to their non-anchoring versions. Combining local administration of IL-12-mouse serum albumin (MSA)-Lumican with a cancer vaccine, CAR-T cell therapy, or neoadjuvant checkpoint blockade improves anticancer effects without exacerbating toxicity. Moreover, this local therapy can prime a protective and systemic CD8^+^ T cell response, leading to curative abscopal effects on non-cytokine-injected tumors^61^.

To enhance the translatability of IL-12 therapy to non-superficial and metastatic tumors, the Hubbell team developed CBD-IL-12, a fusion protein consisting of IL-12 and the A3 domain of VWF. CBD-IL-12 exhibited increased accumulation within tumors and decreased systemic distribution, resulting in profound alterations in the TME. Compared to unmodified IL-12, CBD-IL-12 showed enhanced antitumor activity and reduced systemic toxicity. Mechanistically, CBD-IL-12 activated both innate and adaptive immunity in a metastatic model. Furthermore, CBD-IL-12 can synergize with checkpoint inhibitors and elicit an antigen-specific immune response^62^.

Zou et al.^63^ engineered *α*PD1-mIL12mut2 immunocytokine composed of a low-affinity mouse IL-12 mutant fused to the C-terminus of anti-PD-1. *α*PD1-mIL12mut2 treatment efficiently inhibits tumor growth without toxicity and exerts an abscopal effect on distal tumors and metastasis suppression. This immunocytokine selectively targets tumor-infiltrating PD-1^+^CD8^+^ T cells, enhancing its bioactivity *via* anti-PD-1-mediated *cis*-binding of IL-12 mutant, with a mechanism involving IFN-*γ*^63^.

#### 2.3.2 IL-12-based immunocytokines under clinical research

NHS-IL12 consists of two human IL-12 heterodimers attached to the C-terminus of DNA/histone-binding antibodies called NHS76, which delivers IL-12 to neoplastic lesions by binding to free DNA fragments of histones^64^. In syngeneic mouse models, NHS-IL-12 exhibits significant antitumor effects surpassing non-targeted recombinant IL-12, primarily mediated by CD8^+^ T cells. Combining NHS-IL12 with tumor vaccines, radiotherapy, and chemotherapeutic drugs such as sunitinib, paclitaxel, and gemcitabine synergistically enhances its antitumor effects. Although a phase I clinical study demonstrated the safety of NHS-IL-12 with a maximum tolerated dose of 16.8 μg/kg, no significant antitumor effect was observed in patients^65^.

L19-IL-12 is immunocytokine consisting of IL-12 and a scFv that selectively binds to the alternatively spliced EDB domain of fibronectin. In orthotopic syngeneic mouse models of GL-261 glioblastoma and SMA-560 astrocytoma, L19-IL-12 efficiently targeted the brain tumors, resulting in cure rates of 40% and 29% respectively. Mechanistically, L19-IL-12 can increase the infiltration and activation of CD4^+^ T cells, CD8^+^ T cells, and NK cells in mouse glioma models^66^. Philogen has started a phase I clinical trial in patients with advanced solid tumors that have progressed after immune checkpoint blockade therapy (NCT04471987).

AS1409 is a novel fusion protein comprising a full-length humanized antibody BC1, which targets the EDB isoform, and IL-12. In a phase I clinical trial, a maximum tolerated dose of 15 μg/kg AS1409 per week was determined, accompanied by elevated IFN-*γ* and CXCL10 levels in all patients. During the study, 46% of participants maintained a stable disease state^67^.

### 2.4 IL-21

IL-21 has shown promising antitumor activity in clinical trials by augmenting the effector function of NK cells and T cells, as well as regulating B cells differentiation and survival^68^, ^69^, ^70^. However, its toxicity profile and limited clinical activity make it an attractive candidate for combination therapy and immunocytokine development with antibodies^71^, as listed in Table 5.

**Table 5:** Representative IL-21-based immunocytokines.

Name	Structure	Target	Cytokine	Ref.
*α*CD20-IL-21		CD20	IL-21	72
PD-1Ab21		PD-1	IL-21	73
Erb-IL-21		EGFR	IL-21	74
AMG256		PD-1	• R9E, R76A • Reduced affinity to IL-21R	75
IL-21-*α*HSA		HSA	IL-21	76

#### 2.4.1 IL-21-based immunocytokines under preclinical research

*α*CD20-IL-21, a fusion protein comprising native human IL-21 fused to the NH2 terminal domain of anti-CD20, can directly induces apoptosis in B-cell lymphomas that were resistant to native IL-21 treatment. It also enhances NK cell activation, effector functions, and IFN-*γ* production, resulting in greater ADCC compared with IL-21 and/or anti-CD20 treatments^72^. Furthermore, *α*CD20-IL-21 therapy demonstrated superior tumor control in the rituximab-resistant A20-huCD20 tumors.

PD1-Ab21 consists of a noncovalent homodimeric form of anti-PD-1 scFv and IL-21 at the C-terminus of light chain. It induces differentiation of naïve-like T cells from activated CD8^+^ T cells and promotes the generation of memory stem T cells (T_SCM_) with enhanced cell proliferation^73^. Additionally, PD1-Ab21 treatment showed potent antitumor effects in mice with established tumors, accompanied by an increased frequency of T_SCM_ and robust expansion of tumor-specific CD8^+^ T cells with a memory phenotype. Importantly, PD1-Ab21 outperformed the combination of anti-PD-1 and IL-21 infusion in terms of efficacy.

Erb-IL-21, which is an Erbitux-based IL-21 tumor-targeting fusion protein and features asymmetric structure, demonstrated prolonged half-life and improved antitumor efficacy. Interestingly, Erb-IL-21 demonstrated comparable antitumor efficacy but much lower toxicity than Erb-IL-2. Mechanistically, Erb-IL-21 selectively expanded functional cytotoxic T lymphocytes but not dysfunctional CD8^+^ T cells in the TME^74^. The antitumor effect of Erb-IL-21 primarily depended on existing intratumoral CD8^+^ T cells, rather than newly migrated CD8^+^ T cells. In addition, Erb-IL-21 can synergize with checkpoint blockade in advanced tumors.

#### 2.4.2 IL-21-based immunocytokines under clinical research

Amgen has reported a novel class of immunocytokines fusing an anti-PD-1 antibody and an IL-21 cytokine mutein. Among these, AMG256, fused with IL-21(R9E, R76A), has shown remarkable efficacy in a refractory humanized mouse model resistant to anti-PD-1 monotherapy. Notably, targeted delivery of IL-21 minimized potential detrimental effects on local antigen-presenting cells. Moreover, the decreased affinity of IL-21(R9E, R76A) for its cognate receptors resulted in an extended half-life compared to wild-type counterpart^75^. A phase I trial established to evaluate the safety and tolerability of AMG256 (NCT04362748).

IL-21-*α*HSA (JS014) is actually an engineered long-acting IL-21 by fusing with a nanobody targeting human serum albumin (HSA). This design has dramatically extended half-life and increased exposure of IL-21 in cynomolgus monkeys, with the *t*_1/2_ and AUC approximately 10 and 50 times greater than native IL-21 (*t*_1/2_ (h), 7.08 *vs* 0.81; AUC (μg/mL·h), 36.3 *vs* 0.69), respectively. When combined with PD-1 and T cell immunoreceptor with Ig and ITIM domains (TIGIT) blockades, IL-21-*α*HSA not only augmented antitumor responses but also conferred protection against tumor rechallenge, suggesting the establishment of long-term antitumor immunity^76^. A phase I clinical trial is underway to assess the safety and potential efficacy of JS014 as a single agent and in combination with pembrolizumab in patients with advanced cancer (NCT05296772).

### 2.5 IFNα

IFN*α*, a member of the type I interferon family, holds a pivotal role in regulating the genes that influence tumor growth, proliferation, migration, apoptosis, and differentiation^77^. Being the first approved cytokine drug for tumor immunotherapy, IFN-*α*′s clinical use has long been constrained due to side effects and a short half-life^78^. Recently, IFN*α* has gained prominence in the development of immunocytokines, as listed in Table 6.

**Table 6:** Representative IFN*α*-based immunocytokines.

Name	Structure	Target	Cytokine	Ref.
20-C2-2b		HLA-DR and CD20	IFN*α*2b	80
C2-2b-2b		HLA-DR	IFN*α*2b	81
20-2b		CD20	IFN*α*2b	82
AcTaferon		CD20	• IFN*α*2 (Q124R) • Reduced affinity for IFNAR	83
IFN*α*-anti-PD-L1		PD-L1	IFN*α*	84
Anti-CD20-IFN*α*		CD20	IFN*α*	85

#### 2.5.1 IFNα-based immunocytokines under preclinical research

The earliest reported immunocytokines based on IFN*α* have primarily focused on hematological malignancies, such as 20-C2-2b, C2-2b-2b, 20-2b, and etc.^10^^,^^79^. 20-C2-2b was the first bispecific antibody-IFN*α*2b immunocytokine, comprising two copies of IFN*α*2b and a stabilized F(ab)_2_ of humanized anti-HLA-DR fused to humanized anti-CD20^80^. C2-2b-2b and 20-2b were generated by fusing four IFN*α*2b groups to anti-HLA-DR and anti-CD20, respectively^81^^,^^82^.

Both 20-C2-2b and C2-2b-2b showed toxicity towards B cells, monocytes, and DC cells^81^^,^^82^. To enhance safety, an activity-on-target cytokines called AcTaferon was developed coupling human IFN*α*2 (Q124R), which is approximately 100-fold less active on mouse cells than murine IFN*α*, to a VHH targeting CD20^83^. In mouse models bearing B16-CD20^+^ tumors, AcTaferon demonstrated a comparable therapeutic effect to wild type mCD20-mIFN*α*2, despite being administered at a 1000-fold lower dose.

In addition to their antitumor effects, IFNs are powerful inducers of PD-L1 expression, thus dampening T-cell responses to tumors. To address this, Fu et al. engineered IFN*α*-anti-PD-L1 to induce feedforward responses. This approach synergistically overcame resistance to type I IFN and checkpoint blockade therapy while minimizing side effect in advanced tumors. Intriguingly, tumor regression was mediated by activating IFNAR signaling in host cells rather than tumor cells^84^.

#### 2.5.2 IFNα-based immunocytokines under clinical research

IGN002, an anti-CD20-IFN*α* fusion protein, consists of IFN*α*2b fused to C-terminus of the heavy chain of anti-CD20 *via* a short peptide linker, demonstrated greater antitumor activity in rituximab-resistant tumor models. Mechanistically, the tumor eradication mediated by anti-CD20-IFN*α* requires expression of type I IFN receptor on the tumor cell surface and optimal tumor inhibition requires CD20 targeting^85^. A phase I study is underway to assess safety and efficacy of IGN002 in patients with refractory non-Hodgkin lymphoma (NCT02519270)^86^.

## 3 Unveiling the next Frontier: Novel and promising cytokines/immunocytokines

Advancements in immunological and bioengineering research have recently facilitated the use of immunosuppressive and immunoregulatory cytokines, such as IL-10 and IL-18, in crafting immunocytokines. We will discuss some representative molecules of these cytokines herein.

### 3.1 IL-10

Historically deemed an immunosuppressive cytokine for its dampening effect on pro-inflammatory cytokines like IFN-*γ*, IL-2, TNF-*α*, and GM-CSF, IL-10's role has been redefined by recent studies highlighting its capacity in fostering immune cell proliferation and infiltration in the TME^87^. Pegilodecakin, a PEGylated form of IL-10, activated CD8^+^ T cell immunity in cancer patients by elevating IFN-*γ* and granzyme B levels^88^. Moreover, the IL-10-Fc fusion protein has shown significant potential in enhancing expansion and function of terminally exhausted CD8^+^ TILs through the upregulation of oxidative phosphorylation^89^.

**Figure 2:**
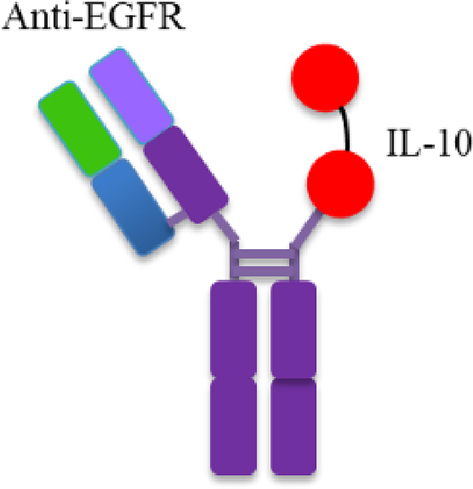
Schematic structure of CmAb-(IL10)_2_. It features asymmetric structure, in which one arm (Fab fragment) is derived from Cetuximab and the other arm is an IL-10 dimer.

The only reported IL-10-based immunocytokine is CmAb-(IL10)_2_, features an arm of Cetuximab replaced by an IL-10 dimer (Fig. 2)^90^. It outperforms its non-targeting IL-10 and Cetuximab counterparts in antitumor efficacy and toxicity profile. Mechanistically, CmAb-(IL10)_2_ prevented IFN-*γ*-induced intratumoral CD8^+^ T cell apoptosis *via* IL-10 receptor signaling of dendritic cells. These findings underscore the significant promise of IL-10-based immunocytokines in cancer therapy.

### 3.2 IL-18

IL-18 has demonstrated significant antitumor potential. A phase I clinical trial highlighted IL-18's immunomodulatory activity, however, a subsequent phase II clinical trial did not confirm substantial antitumor activity^91^^,^^92^. The presence of IL-18 binding protein (IL-18BP), which has a higher affinity for IL-18 than IL-18R*α*, impedes IL-18 binding to its receptors, acting as a secretory immune checkpoint in the TME and thus limiting its therapeutic efficacy^93^. To overcome this, DR-18, a mutated form of IL-18, has been engineered to avoid neutralization by IL-18BP while maintaining affinity for IL-18R*α*. DR-18 exerts potent anti-tumor effects by promoting the development of poly-functional effector CD8^+^ T cells, diminishing T cells exhaustion, and expanding the pool of stem-like TCF-1^+^ precursor CD8^+^ T cells^94^. Although there are no reports of IL-18-based immunocytokine, IL-18 and its mutant DR-18 hold great promise for future of immunocytokine development.

### 3.3 IL-24

IL-24 is recognized for its ability to suppress tumor invasion, metastasis, and the stemness of tumor stem cells^69^. Clinical studies correlate decreased IL-24 expression with tumor progression and a poor prognosis. In a phase I trial, intra-tumoral injection of Ad-mda-7, an adenovirus vector encoding IL-24, induced extensive apoptosis in tumor cells, yielding substantive clinical benefits in 44% of participants^95^. IL-24's antitumor effects are also attributed to its capacity to effectively reduce vascular endothelial growth factor levels, stimulate CD4^+^/CD8^+^ T cell, and alter the TME into a more immune-stimulatory milieu^96^. Furthermore, IL-24 in combination with other treatments, such as EGFR inhibitors, Her2 inhibitors, radiotherapy, and cell therapy, demonstrates enhanced synergistic antitumor effects^96^. IL-24's multifaceted role underpins its promise as a therapeutic cytokine. The construction of antibody-cytokine fusion proteins that target IL-24 at the tumor site holds significant potential in overcoming resistance and immune evasion.

### 3.4 TNF-α

**Figure 3:**
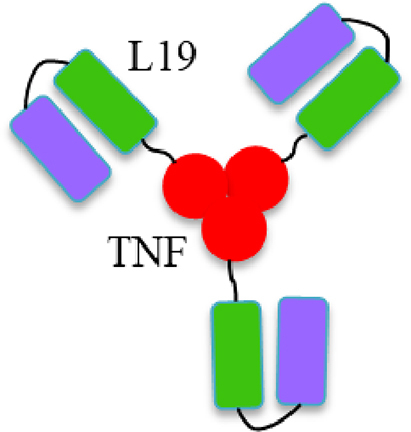
Schematic structure of L19-TNF. The homotrimeric fusion protein L19-TNF consists of the L19 antibody, in scFv format, fused to TNF.

In addition to the aforementioned recently identified cytokines, TNF-*α* holds a unique position in the development of immunocytokines, but its application remains controversial. Despite being one of the earliest cytokines investigated in this field, only one product, L19-TNF (Fig. 3), has advanced to clinical stages^97^. Initially recognized for its cytotoxic properties, TNF-*α* is now understood to also have an immunosuppressive function, promoting the accumulation and/or activity of Tregs, regulatory B lymphocytes (Bregs), as well as MDSCs^98^. This duality poses challenges for the future development of TNF-based immunocytokines.

## 4 How to address the safety challenges: Next-generation immunocytokine

Immunocytokines are hindered by the “cytokine sink” effect where they are trapped by cognate receptors in circulation before reaching their target tumor sites. Moreover, studies have reported that only a minimal portion of immunocytokine accumulates in neoplastic lesion (in the best cases, 0.01%‒0.1% injected dose/gram of tumor), leading to toxicity similar to the parental cytokine^83^^,^^99^. To address the “cytokine sink” and improve tumor targeting, research is mainly focused on two strategies: using cytokine mutants with reduced receptor affinity or creating conditionally activated immunocytokine prodrugs.

### 4.1 Immunocytokines fused with potency-reduced cytokines

**Figure 4:**
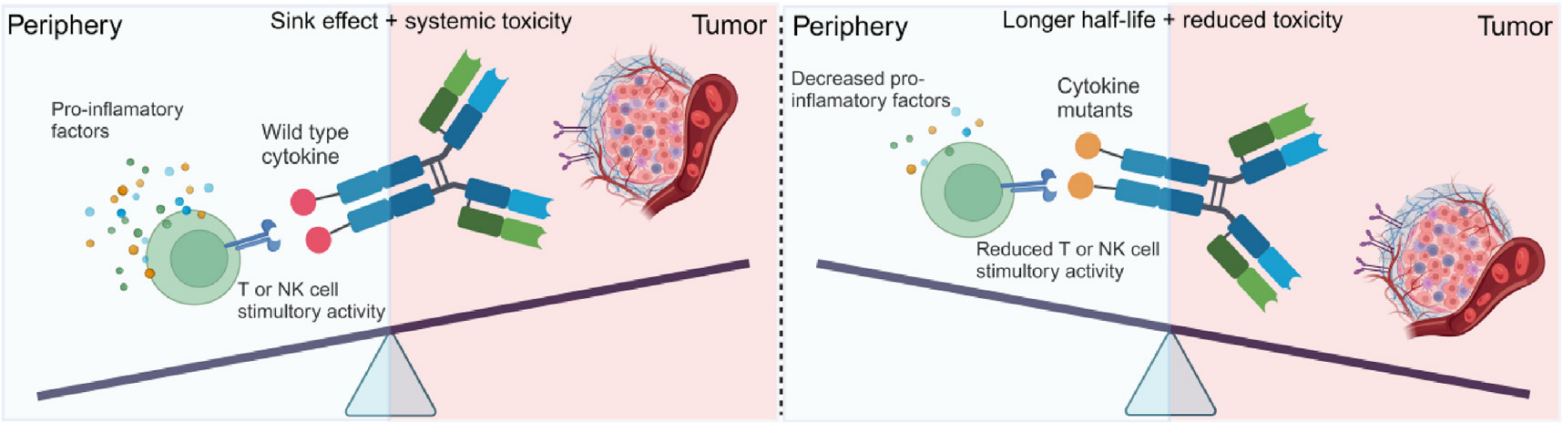
Schematic of action model of wild-type immunocytokines and mutant immunocytokines. The wild-type immunocytokines can be neutralized by cytokine receptors in the peripheral lymphocytes, leading to a shortened half-life and heightened toxicities. The mutant immunocytokines, characterized by reduced affinity for cognate receptors, demonstrate diminished side effects and an increased propensity to infiltrate tumor tissues, thereby enhancing their therapeutic effects.

Reducing cytokine affinity towards the cognate receptor can mitigate the “cytokine sink” effect, thereby extending half-life and enabling antibody-directed tumor accumulation. In addition, decreased biological activity can elevate drug amounts at the tumor site due to prolonged half-life and allow the use of higher doses of immunocytokines^99^ (Fig. 4). Mutants of cytokines such as IL-2, IL-15, and IFN*α* have shown enhanced druggability of related immunocytokines^43^^,^^100^^,^^101^. Recently, the development of anti-PD-1-based immunocytokines has opened up new avenues. PD-1 *cis*-targeted IL-2/IL-15R agonists, such as PD-1/IL-2v, PD-1-laIL-2, *α*PD1-IL15m, and *α*PD1-IL15-R, can selectively deliver the attenuated IL-2 or IL-15 to PD-1^+^CD8^+^ TILs, bypass NK cells, and exert significantly antitumor activity in multiple tumor models^30^^,^^35^^,^^43^^,^^44^.

### 4.2 Conditionally-activated immunocytokine prodrugs

**Figure 5:**
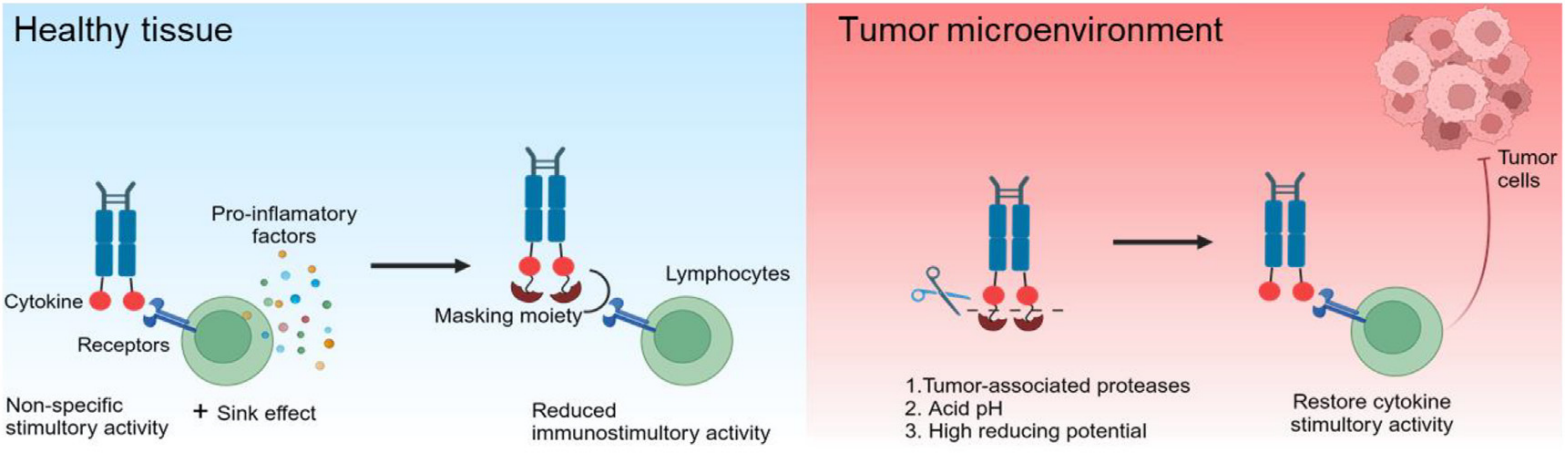
Schematic of the tumor-conditional cytokine prodrug in healthy tissue and tumor site. In healthy tissue, cytokine is shielded by the masking moiety; in the tumor, it is cleaved by tumor-associated proteases, redox, or acid pH, releasing the immunostimulatory cytokine.

Prodrug-based strategies offer a potential approach to improve the safety profile of immunocytokines while maintaining antitumor activity (Fig. 5). Leveraging tumor associated proteases is a promising direction for achieving tumor-localized cytokine activation^102^. Various masking domains, such as native cytokine receptors, antibody fragments, peptides, and polyethylene glycol (PEG), have been used to shield cytokines. The Fu group has reported cognate receptor-masked IL-2, IL-12, IL-15, and IFN*α* prodrugs^103^, ^104^, ^105^, ^106^. WTX-124, an IL-2 prodrug consisting of native human IL-2 linked to a Fab antibody fragment (inactivation domain) and a single-domain antibody targeting human albumin (half-life extension domain), is in phase I clinical trial (NCT05479812) by Werewolf^107^. The Tang group utilized biocompatible polymers PEG to mask IL-15 through chemical linkers that are responsive to tumor-specific stimuli, such as high reducing potential and acid pH, which led to equivalent antitumor efficacy to the parental cytokine with markedly reduced toxicities^108^.

Currently, limited research has been conducted on immunocytokine prodrugs. AskGene has reported an anti-PD-1/IL-15 prodrug, ASKG915, which utilizes cognate IL-15R*β* to mask the activity of IL-15^109^. Additionally, our team has developed a tumor-conditional anti-PD-L1/IL-15 immunocytokine (LH05), which innovatively employs steric hindrance of its flanking moieties of anti-PD-L1 and IL-15R*α*-sushi domain to mask IL-15^110^.

### 4.3 Silencing Fc functions of antibody by point mutations

The toxicity of immunocytokines is mainly attributed to their cytokine portion, while their antibody component also has potential implications for safety. Target-antigen may be expressed on normal cells and contribute to on-target off-tumor binding of immunocytokines^11^. Moreover, receptors which bind conserved Fc regions of IgG antibodies such as Fc gamma receptors (Fc*γ*Rs), neonatal Fc receptor (FcRn) and C-type lectin receptors (CLRs) may also contribute to target-independent trafficking of immunocytokines in normal cells^111^. It is considered that antibody aggregates have potential risk for activating Fc*γ*Rs on immune cells, which could exhibit off-target toxicity. It has been shown that the Fc*γ*R-mediated off-target cytotoxicity of antibody-drug conjugate aggregates can be reduced by engineering Fc (L234A/L235A mutant) for silencing Fc-mediated effector functions, which also has significant implications for enhancing the safety of immunocytokines^112^.

In terms of immunocytokines, the primary role of their antibody component is to serve as a missile to deliver the cytokine payload to target cells or neoplastic sites. Therefore, sometimes it may need to reduce Fc-mediated effector functions, especially for anti-PD-1/PD-L1-based immunocytokines. For example, KD033, a L234A/L235A modification was incorporated into its Fc region to minimizing ADCC^46^; LH01, a N297A mutation was incorporated into its Fc region to remove ADCC^41^^,^^113^.

### 4.4 Optimizing the selection and combination of antibodies and cytokines

The anti-PD-1/IL-15 immunocytokine, as reported by Fu team, induced the complete demise of MC38 tumor-bearing mice when administered intraperitoneally twice at a dosage of 30 μg^44^. Conversely, our team's anti-PD-L1/IL-15 immunocytokine (LH01), possessing a similar homodimeric structure with the anti-PD-1/IL-15, demonstrated no notable toxicities upon intraperitoneal administration three times at a dosage of 5 mg/kg (100 μg) in MC38 tumor-bearing mice^41^. This suggests that fusing antibodies targeting different antigens with the same cytokine can markedly impact the safety of the constructed immunocytokines. Additionally, the CmAb-(IL10)_2_, an anti-EGFR/IL-10 immunocytokine, showed good safety profile at a dosage of 4 mg/kg. In contrast, the anti-EGFR/IL-2 immunocytokine (CmAb-IL2) induced body weight loss of mice at a dosage of 0.8 mg/kg, and all mice treated at a dosage of 2 mg/kg died^90^. This indicates that fusing the same antibody with different types of cytokines can also lead to varying levels of side effects. It's worth noting that beyond safety concerns, the combination of different antibodies with various cytokines can significantly affect the antitumor efficacy. For instance, the anti-tumor effect of *α*PD-1-IL-15-R, which targets PD-1, is more potent than that of *α*EGFR-IL-15-R, which targets EGFR^44^. Overall, in constructing immunocytokines, it's imperative to align with their mechanisms of action and subsequently fine-tune the combination of antibodies and cytokines. This approach is paramount for enhancing the safety profile of immunocytokines and maximizing their anti-tumor efficacy.

## 5 Summary and future perspectives

### 5.1 Optimizing antibody and cytokine selection for immunocytokine construction: Ensure superior safety and efficacy than the combination therapy

To enhance cytokine targeting delivery, antibodies targeting tumor-associated antigens (such as FAP, EDB, EGFR, CBD, and integrin), PD-L1, and PD-1 have been employed. Meanwhile, cytokines such as IL-2, IL-10, IL-12, and IL-15, have shown immense potential for fusion with antibodies^2^^,^^13^^,^^102^. How to “match” antibodies and cytokines requires careful consideration. An essential criterion is that immunocytokines should demonstrate superior antitumor activity and safety compared to the combined administration of antibodies and cytokines. For example, PD-1-cis-immunocytokines have the advantage of selectively delivering IL-2 or IL-15 to PD-1^+^CD8^+^ T cells while bypassing non-targeted cells, thereby activating intra-tumoral effector CD8^+^ T cells in a more targeted manner.

### 5.2 Improving immunocytokine safety: Leveraging cytokine engineering and prodrug strategies

To improve the safety profile of immunocytokines, one strategy involves engineering cytokines with lower natural receptor affinity, as seen with IL-2, IL-15, and IFN*α* mutants^100^, ^101^, ^102^. However, these mutants reduce their affinity for both intratumoral and peripheral lymphocytes, presenting a challenge to the balance between insufficient antitumor activities at low doses and the risk of systemic toxicity at high doses^99^. An alternative is to employ prodrug techniques that allow the cytokines to be conditionally activated within the TME, utilizing tumor-specific properties like proteases, pH, and redox potential for targeted cytokine activation^102^^,^^108^^,^^114^. However, individual differences in tumor-associated properties may introduce uncertainties in the clinical applications of such products, which should be considered in all prodrug strategies. Furthermore, the choice of masking strategy is of great importance. Despite the cognate receptor-masked strategy can reduce the peripheral activity of the cytokine, the introduction of the masking receptor complicates the molecular structure, thereby increasing the risk of immunogenicity^110^. Although speculative, a combined approach, integrating affinity attenuation with prodrug strategies, might provide a pathway to immunocytokines with optimized safety, effectiveness, and targeted activation.

### 5.3 Combination therapy: Enhancing the antitumor potential

The combination of drugs with different mechanisms of action often yields improved antitumor effects. Immunocytokines offer advantages over the combined use of antibodies and cytokines, making them promising candidates for co-administration with other therapeutics, including antibodies, chemotherapy, radiation therapy, and adoptive cell transfer (ACT) therapy, etc. For instance, combining PD-1/IL-2v and anti-PD-L1 can break tumor immunity resistance by enhancing stem-like tumor-reactive CD8^+^ T cells and reprogramming macrophages^115^. Moreover, the anti-PD-L1/IL-15 immunocytokine prodrug LH05 can synergize with oncolytic poxvirus to induce stronger antitumor activity resulting from it enhancing the effector function of CD8^+^ T cells rather than increasing CD8^+^ T cell infiltration^110^. A combination of anti-CD20-IL-2 immunocytokine with CD19-specific T cells improved *in vivo* T-cell persistence, which led to an augmented clearance of CD20^+^CD19^+^ tumor beyond monotherapy^116^. The efficacy and safety profile of N-803 plus BCG exceeds that of other available intravesical and systemic options for BCG-unresponsive NMIBC, which indicates that combining immunocytokines with therapeutic vaccines could hold significant clinical promise^117^. In addition, given IL-15's pivotal role in NK cell immunity, the combined application of IL-15-based immunocytokines with NK cell therapies also holds immense promise^118^^,^^119^.

### 5.4 Immunocytokines carrying different payloads: Playing pleiotropic antitumor effects

Both preclinical and clinical studies indicate that combining various cytokine products with different cytokines payloads demonstrates synergistic antitumor effects^120^, ^121^, ^122^. However, the determination of the optimal dose for product combinations represents a complex multidimensional clinical challenge^123^. Therefore, the use of immunocytokines with two cytokine payloads could potentially simplify clinical development^124^^,^^125^. IL-2/TNF, IL-2/TRAIL, IL-2/IL-12, and other dual cytokine-antibody fusion proteins have shown promising development prospects^124^, ^125^, ^126^, ^127^. Optimizing the relative level of each cytokine has proven crucial for attaining synergistic effects, given the varied pharmacological properties and half-lives of cytokines^124^. When developing dual cytokine-antibody fusion proteins, such considerations are essential. For instance, due to differing potency, the dosage of IL-2 fusions are typically over 10-fold that of TNF fusions^12^. To address this, Neri team identified a TNF mutant with reduced activity, resulting in the construction of a potency-matched dual-cytokine-antibody fusion protein, named IL-2-F8-TNF^mut^. In tumor-bearing mice, IL-2-F8-TNF^mut^ demonstrated superior antitumor effects compared to the wild-type counterpart^127^^,^^128^.

In conclusion, although immunocytokines are currently in the early stages of research, their superiority is anticipated to be recognized and wildly implemented in clinical practice in the future. We hope that our review provides insightful guidance and references for the design and development of immunocytokine-based therapeutics.

## Author contributions

Wenqiang Shi and Nan Liu designed and wrote the paper and prepared figures. Huili Lu edited and revised the manuscript.

## Conflicts of interest

The authors declare no conflicts of interest.
